# Mapping Perceived Impact, Facilitators and Barriers of Cystic Fibrosis Management in Children and Adolescents: A Qualitative Study From the Parents' Perspective

**DOI:** 10.1111/scs.70106

**Published:** 2025-09-10

**Authors:** Sandra Gagulic, Ana Bártolo, Teresa Reis Silva, Raquel Penteado, Alda Marques

**Affiliations:** ^1^ Centro de Investigação em Atividade Física Saúde e Lazer (CIAFEL) da Faculdade de Desporto da Universidade do Porto (FADEUP) Porto Portugal; ^2^ Insight: Piaget Research Center for Ecological Human Development Instituto Piaget‐ESS/Piaget Viseu Portugal; ^3^ CINTESIS@RISE, CINTESIS.UPT Portucalense University Porto Portugal; ^4^ Cystic Fibrosis Reference Center, Hospital Pediátrico de Coimbra Unidade Local de Saúde de Coimbra Coimbra Portugal; ^5^ Lab3R – Respiratory Research and Rehabilitation Laboratory School of Health Sciences (ESSUA) and Institute of Biomedicine (iBiMED), University of Aveiro Aveiro Portugal

**Keywords:** adherence, chronic respiratory disease, cystic fibrosis, family caregivers, paediatric

## Abstract

**Background:**

Cystic fibrosis imposes a significant treatment burden on children and their informal caregivers, who have to change their routines to carefully adhere to medication and exercise as treatment regimes. Although informal caregivers are known to be key players in the daily management of these children, their own voice is scarcely explored, often hindering personalisation of care. The main objective of the study was to map the multifactorial impact of cystic fibrosis, as well as identify barriers and facilitators perceived by parental caregivers in managing the disease in the paediatric age.

**Methods:**

A qualitative exploratory study was conducted involving six fathers and 14 mothers of 15 children and adolescents (6–18 years; 40% male) diagnosed with cystic fibrosis. Semi‐structured individual interviews were conducted. Data were analysed using inductive thematic analysis.

**Results:**

Four main themes emerged from the analysis: (1) perceived impact of the disease; (2) facilitators of disease management; (3) adherence to treatment; and (4) physical activity. Findings emphasised the emotional impact of the disease, especially when diagnosed at a later stage of development. Increased knowledge seemed to facilitate adaptation and daily management, as well as a normalisation of attitudes by parents. All parents recognised physical activity as an important part of treatment, although financial and logistical factors (e.g., reconciling schedules) were important barriers to adherence.

**Conclusions:**

Our findings suggest that disease management and specifically adherence to treatment recommendations is impacted by early diagnosis, attitudes towards the disease, social support and financial constraints. Future interventions should focus on identifying the needs and supporting the whole family to cope with the demands of the disease, namely by improving knowledge about the benefits of different intervention approaches.

## 1 Introduction

Cystic fibrosis (CF) is a rare multifactorial genetic disease [1]. According to the European Cystic Fibrosis Society [2], 54,043 cases of CF have been registered in Europe. Of these, 46% are children with an average age of diagnosis of three and a half months. Despite the advances in understanding the disease and its associated therapies resulting in individuals' lung function and survival improvements [3], quite complex and time‐consuming treatments need to be undertaken on a daily basis [4].

Parents assume, in most cases, the role of main informal caregivers [5] and have to deal with the demands of adhering, delivering and supervising CF‐specific therapies, medical appointments, hospital admissions, financial difficulties and uncertainty about their children's future [6, 7]. Taken together, these aspects add more stress to their roles and increase conflict with adolescents [8]. Moreover, children and adolescents deal daily with the need to manage the challenges of disease and developmental transitions [9]. Children and parents are, therefore, both at a considerable risk of developing psychological symptoms, such as depression and anxiety, and a poor quality of life as a result of these responsibilities [9, 10].

Recent research has indicated an association between depressive symptoms in children with CF and their parents and low confidence in medical treatments, which may have an impact on treatment compliance [11]. Adherence to physical exercise in CF is not always considered a priority by family caregivers [12, 13, 14], although it is currently seen as a crucial part of integrated CF treatment [15]. Thus, non‐adherence to the different CF intervention regimens can have serious implications for children's health, leading to an increase in respiratory exacerbations, hospitalizations and loss of lung function, which are leading causes of premature death related to the disease [16]. It is, therefore, important to understand the perceived impact of the disease from the caregiver's perspective, playing a fundamental role at this stage of development, and the factors that affect management and consequent adherence to different medical recommendations, such as medication and physical exercise.

Several studies have already focused on understanding parents' perceptions and experiences of the physical and emotional impact of CF [5, 7, 9], as well as the changes caused in family dynamics [17]. However, to date and to the best of our knowledge, the barriers and facilitators of disease management and the reasons for non‐adherence to an integrated treatment regimen, from the perspective of caregivers, are largely unexplored. This knowledge can contribute to identifying factors that should be targeted when personalising the interventions to improve disease management and adherence in this context. The main objective of the study was to map the multifactorial impact of CF, as well as identify barriers and facilitators perceived by parental caregivers in managing the disease in the paediatric age.

## 2 Methods

### 2.1 Study Design and Ethics

An exploratory qualitative study was carried out to map the multifactorial impact of CF from parents' perspective [18]. This study is a subset of a larger project which adhered to the Declaration of Helsinki [19] and has received ethics committee approval. All participants were informed that their involvement in the study was voluntary and confidential. Written informed consent was obtained before any data collection. This study is reported in accordance with the Consolidated Criteria for Reporting Qualitative Research (COREQ) checklist [20] to ensure transparency, rigour and comprehensiveness of the study context and its findings.

### 2.2 Research Team and Reflexivity

Three authors were involved in the analytical process to ensure trustworthiness, confirmability (i.e., researcher objectivity) and reflexivity. The team's diverse expertise and professional backgrounds informed the authors' pre‐understandings, directly influencing decisions from the selection of the research topic to data interpretation. The first author, a physiotherapist and PhD student with extensive clinical experience in CF, contributed to a practical, patient‐centred perspective that guided the development of the interview guide and facilitated a sensitive approach to data collection. This expertise necessitated reflexivity to mitigate potential bias stemming from her familiarity with CF care. The second author, an experienced qualitative researcher with a PhD, systematically reviewed transcripts and contributed to the analytical and interpretive phases. Her expertise in qualitative methodologies ensured analytical rigour, facilitating robust theme identification and balancing SG's clinical perspective with methodological objectivity. The third author, a PhD researcher specialising in respiratory health, reviewed thematic coding and engaged in interpretive discussions with other authors. Her scientific background in respiratory care brought depth to the CF‐specific analyses while upholding scientific rigour. The absence of pre‐existing relationships between researchers and participants minimised interpersonal bias. Through regular reflexive discussions, the team critically engaged with their pre‐understandings, reducing personal influence on the analytical process. Each researcher's unique expertise enriched the interpretive framework, strengthening both the depth and validity of the study's findings [21].

### 2.3 Participants and Recruitment

Recruitment was performed in a Paediatric hospital in Portugal, using a purposive sampling technique. A subset of parents included in a larger project aiming at investigating the effects of a home‐based exercise intervention to CF children that accepted to participate was included in this study. Children were eligible for inclusion in the study if they (i) were 18 years of age or younger; (ii) were not mechanically ventilated; (iii) had a respiratory function test result of FEV^1^ > 40 [22]; and (iv) regularly attended their child's hospital appointments. All parents whose children had a current acute respiratory exacerbation identified by an attending physician were excluded. Clinicians at the institution identified and contacted eligible individuals, explained the purpose of the study, checked their willingness to participate, and referred those interested to take part to the main researcher. All parents of individuals that met the inclusion criteria accepted to participate in the study.

### 2.4 Data Collection

In‐depth individual interviews were conducted to explore the perceptions of parents of children or adolescents diagnosed with CF. A semi‐structured interview guide with open‐ended questions was developed and pilot tested with two caregivers before collecting data. After small adjustments in the language, the questions focused on mapping parents' perceptions of the impact of the disease on their child's life and their own, such as daily management and adherence to treatment, as well as their relationship with physical activity (Table 1). A researcher trained in qualitative research conducted the interviews face‐to‐face in a private room at the hospital. Field notes were taken to register any observations or non‐verbal responses of participants during the interviews which could contribute to informing future data analysis. The interviews lasted between 20 and 60 min, were audio‐recorded (Tascan DR‐05X; TEAC America Inc., Santa Fe Springs, CA) and transcribed verbatim.

**TABLE 1:** Main questions included in the semi‐structured interview script directed to parents of children/adolescents with cystic fibrosis.

Question number	Interview script
1	What impact does the disease have on your family?
2	How do you manage time to care for your child?
3	Do you mind sharing with me the organisation of your day?
4	What do you think about your child's medical condition?
5	What is your opinion on your child's possible participation in sports or other activities involving physical activity?
6	What is your child's opinion of physical activity? Any favourite? If yes, why?

### 2.5 Data Processing and Analysis

**FIGURE 1:**
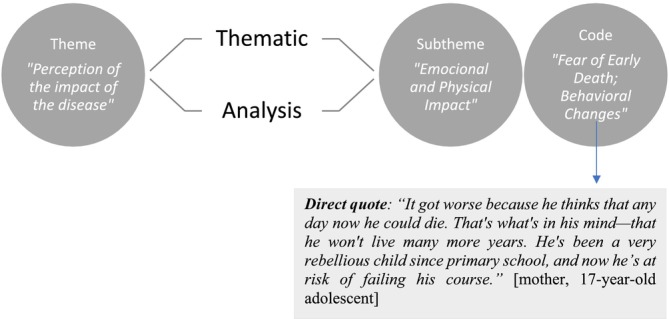
Data analysis process example: thematic analysis.

Data were analysed using an inductive thematic analysis grounded in constructivist epistemology, which asserts that knowledge is co‐constructed through participants' subjective experiences. Themes and sub‐themes were derived directly from these experiences, with codes assigned to relevant segments of text (Figure 1). Two researchers independently analysed the data, following Braun & Clarke's six‐step procedure: (i) familiarisation with the data, (ii) initial coding, (iii) theme identification, (iv) theme review, (v) theme definition and naming, and (vi) reporting [21]. This structured approach allowed for reflexive interpretation of participants' narratives. The ATLAS.ti software (version 22) facilitated the organisation of codes into potential themes, ensuring rigorous analysis aligned with our research objectives. To enhance the study's rigour, we followed Lincoln and Guba's guidelines [23]. Credibility was sought by using triangulation, where multiple researchers analysed and discussed themes to reach consensus. Transferability was achieved via purposive sampling to ensure diverse representation across demographic variables. Dependability was reinforced through regular team discussions and systematic coding processes, while confirmability was ensured through transparent documentation, reflective journaling, and an audit trail. Data saturation was defined as the point where additional data ceased to yield new themes [24]. By situating our analysis within a constructivist framework, we emphasised that participants' realities and social contexts are crucial for understanding the complexities of managing CF, enriching our findings and contributing to a deeper comprehension of the lived experiences of parental caregivers.

## 3 Results

### 3.1 Participants' Characteristics

Thirty‐two eligible individuals were identified. Of these, 20 (14 mothers and six fathers) of 15 children were available to take part in the study and be interviewed; in some cases, both parents of the same child were interviewed. Their children were between 6 and 18 years old (children: 6–8 years [*n* = 6]; adolescents: 10–18 years [*n* = 9]). Parents were on average 38 years old (38 ± 5); most were married (90%; *n* = 18), had secondary education (65%; *n* = 13) and were employed (80%; *n* = 16).

### 3.2 Qualitative Findings

**FIGURE 2:**
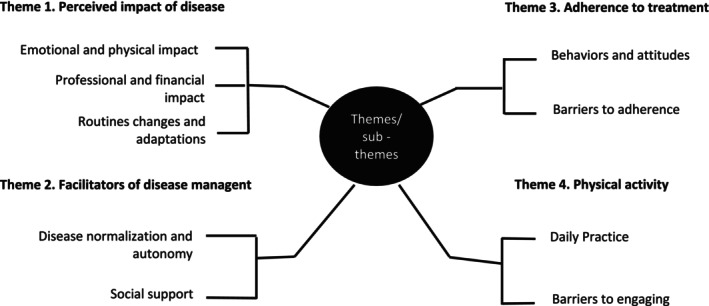
Themes and sub‐themes obtained from the qualitative data analysis (*n* = 20).

Figure 2 shows the themes and sub‐themes that emerged from the thematic analysis. Four main themes emerged: (i) perception of the impact of the disease; (ii) facilitators of disease management; (iii) adherence to treatment; and (iv) physical activity. A summary of these findings is presented below, followed by the most illustrative quotations.

#### 3.2.1 Theme 1: Perception of the Impact of the Disease

Caregivers reported that the period before diagnosis was the phase of greatest impact and family disorganisation. Most parents had no prior knowledge of the disease. In some cases, difficulties were reported in getting the right diagnosis (sometimes mistaken for asthma), which seems to have had a significant emotional impact. For instance, one mother shared: ‘We didn't know (…) we fed her, but because of the illness she lost weight (…) and they said we didn't feed her…’ [mother, 6‐year‐old child]; This perception of misjudgement created feelings of guilt and frustration among caregivers, exacerbating their emotional struggles. Another caregiver highlighted her son's physical exhaustion, explaining: ‘My daughter was coughing so much that tears were running down her face. She wasn't crying. It was the coughing, the effort (…). We had a traumatic time until we got the diagnosis’ [mother, 11‐year‐old adolescent]. Such accounts underscore the prolonged negative impact that both parents and children endured as they struggled with persistent symptoms and sought clarity, ultimately amplifying the emotional challenges they faced in the absence of a clear diagnosis.

Hospital admissions, associated with periods of exacerbation of the child's and/or adolescent's symptoms, were pointed out as major factors of negative emotional impact and with significant implications for family and professional management. Some mothers reported having to quit their jobs to support their children, especially the younger ones: ‘I was working and I had to quit because they didn't want to look after my son at nursery school because of the medication and all that stuff…’ [mother, 6‐year‐old child]: ‘… I'm a beautician, and I had my own office, but when they told me to wait three months at home, one in hospital, there's no one who can keep a business open (…)’ [mother, 11‐year‐old child]. The demands related to frequent trips to the hospital were also highlighted, which meant changes to the daily routine (e.g., professional flexibility, reorganisation of other parental tasks), as well as a greater financial burden: ‘(…) But there was a phase when the hospital allowed us to take the medication home for three months. But then, at some point, they banned it, which forced me to travel here [to the hospital] often to get the medication’ [mother, 18‐year‐old adolescent]. This is suggestive of the continued pressure on families as they overcome logistical and financial hurdles to secure necessary medical care for their children, emerging as a double burden to the already demanding role of caregiver.

From the perspective of most parents, their children were well adapted to the disease, especially when the diagnosis occurred at an early stage of development. Most maintained a daily routine similar to their peers, although they did report greater during some activities or loss of appetite: ‘She feels a bit tired. And if she gets too tired, if she eats, she can throw it all out’ [mother, 6‐year‐old child]. The main impacts, especially on an emotional level, were more noticeable in adolescents diagnosed later, with parents reporting symptoms of hopelessness and challenging behaviour at school: ‘It's gotten worse, because he thinks that from “today to tomorrow” he could die. That's what's in his head. That he won't live for many years. He has been a very rebellious child since elementary school and now he is at danger of failing his course.’ [mother, 17‐year‐old adolescent]. Older children are thus more vulnerable to psychological distress and behavioural changes, which also impact family dynamics.

#### 3.2.2 Theme 2: Facilitators of Disease Management

Parents' reports suggested that the passage of time and the consequent increase in knowledge about the disease led to better adaptation and acceptance by themselves and their children. Parents of adolescents diagnosed in early childhood highlighted their children's autonomy in managing symptoms and therapies, which seemed to help ease the burden on parents and normalise family routines. For instance, one father shared, ‘She has a symptom, gets up, coughs, vomits and that's it’ [father, 16‐year‐old adolescent]; while other mothers similarly expressed confidence in their daughters' abilities to manage their medication: ‘I trust her with the medication. For example, when she eats at school she takes it, I know she doesn't hide it.’ [mother, 11‐year‐old adolescent]; ‘She knows she has to take her medication, she knows the care she has to take’ [mother, 18‐year‐old adolescent]. This sense of independence in children allowed parents to feel more secure about their child's care and facilitated a greater balance within family life.

In addition, the social support network (e.g., family, friends, health professionals) was shown to be an important facilitator in managing the illness, especially for parents. However, conflicting perceptions existed regarding the support provided by formal structures such as the school. Some parents reported an understanding attitude from teachers and educators, while others pointed to this as an additional stress factor: ‘(…) we don't have any support at school for her, because she misses a week, but nobody tells her, take this to make up for it” [mother, 16‐year‐old adolescent].’ These mixed experiences suggest that while informal support networks were generally effective, formal support systems could either mitigate or amplify parental challenges, depending on the level of cooperation provided.

Finally, an encouraging and normalising attitude from parents also appeared to have a positive impact on the children and adolescents dealing with their chronic condition: ‘Our initial goal was to create a sense of normalcy while safeguarding the extra care and activities they have to accomplish. As they grow up, we now strive to ensure that they lead perfectly normal lives’ [mother, 16‐year‐old adolescent]; ‘She has never been a girl who has rebelled against her illness, she takes everything very calmly and always with a smile’ [father, 16‐year‐old adolescent]. These perspectives reinforce the value of normalisation strategies, which can strengthen children's resilience and contribute to their overall well‐being as they face health challenges.

#### 3.2.3 Theme 3: Adherence to Treatment

Parents reported adherence to treatment by children and adolescents as being good, however, a later diagnosis also appeared to be a barrier to adherence, particularly to the use of airway clearance devices such as ‘Flutter’. Emotional factors, such as hopelessness due to the health condition, was pointed out as a contributor for this behaviour: ‘Bad, bad and continues to deal badly, doesn't want to do the flutter, every day is a massacre. (…) I have to force him every day. He says it's not worth doing because he's going to die’ [mother, 17‐year‐old adolescent]. Reports also seemed to suggest that the period of adolescence may lead to more feelings of shame and conditional use of medication in social contexts. Moreover, a negative reaction from peers may further aggravate the child's behaviour and their adherence to medical recommendations: ‘She talks more about the kids… They make fun of her when she wants to expel it. Sometimes she swallows it if she can’ [father, 8‐year‐old child]. These insights suggest that the complexities of adolescence, coupled with emotional struggles and social dynamics, significantly influence treatment adherence.

#### 3.2.4 Theme 4: Physical Activity

All parents reported that their children practised some kind of physical activity; however, the frequency of these activities depended on financial and logistical factors. Most children practised sports activities such as swimming (the most common on medical advice), ballet, and/or soccer; however, families with a greater financial burden only reported involvement in more structured activities carried out at school (e.g., physical education classes). Free activities such as jumping rope and cycling were commonly reported by parents of young children.

Among the main barriers to participation in physical activities, parents highlighted the difficulty of reconciling schedules due to family and/or professional demands, the overload of school activities (especially for adolescents) and the weather conditions in outdoor activities ‘He says he loses a lot in training and has to do his homework, which then affects his school’ [mother, 10‐year‐old adolescent]. Parents' reports also showed that children and adolescents have difficulties in managing activities and the associated tiredness: ‘I think he does too much physical exercise, sometimes blood appears in his secretions and that's why I don't insist too much either and I've been walking with him, so he doesn't play ball so much and run around so much’ [father, 16‐year‐old adolescent]. Finally, another important aspect for managing physical activity seemed to be the involvement of parents in the dynamics, especially for more dependent children. Lack of engagement can discourage exercise: ‘She asks to go for a walk at night, but the person wants to get home and rest a little and doesn't feel like doing anything’ [mother, 6‐year‐old child]. These findings suggest that while physical activity is valued by families, various obstacles—including financial constraints, scheduling conflicts, and parental involvement—play significant roles in shaping children's engagement in exercise. Addressing these barriers may require a multifaceted approach that considers not only the children's needs but also the logistical and emotional support systems within the family.

## 4 Discussion

Our findings suggest that managing CF in children is demanding and has a multidimensional impact, affecting the emotional, physical, and social well‐being of children and their informal caregivers. Overall, participants identified different modifying factors that affected the management of the disease and adherence to an integrated treatment, directly related to the child's characteristics and perceptions and attitudes (e.g., normalisation) towards the disease in the family context. According to the reports, knowledge, the child's greater autonomy in medication management, and social support emerged as main factors that facilitate adherence to therapeutic recommendations. This study confirms earlier research [4, 10, 25] indicating that parents of children diagnosed with CF face significant emotional and job‐related challenges. Hospitalisations due to exacerbations particularly disrupt family life and cause emotional distress. The potential for a terminal illness and various stressors like fear, limited social time, and financial strain significantly affect caregivers' quality of life [5].

Children with chronic illnesses, as previously reported [26], often face higher educational demands compared to their healthy peers, being absent from school an average of 16 days yearly versus 3 days for healthy peers [27]. Our study corroborates these findings, highlighting school support as a key factor in managing these challenges, according to parents.

Late diagnosis exacerbates symptoms and family disruption, hindering adaptation and treatment adherence due to shame and hopelessness about the future.

In fact, early CF diagnosis provides the opportunity to improve illness management and avoid early repercussions by adopting more peer‐like habits [28]. Our study supports findings from earlier literature showing parents preferring an early diagnosis, even when their child has an incurable disease [29]. The majority of kids are better able to accept daily routines and cognitively adjust to changes with a posture of control and normality when they receive an early diagnosis [30].

In addition, families appear to be still challenged by the need to make daily choices about what to prioritise in terms of CF management (academic vs. therapeutic). For example, the inclusion of physical exercise in the routine, as part of the treatment of the disease, was recognised as a benefit for the management of CF symptoms, as the literature has shown, but uncertainty and concern about overdoing it were also reported. Adolescents with greater autonomy also seem to experience this pressure, with the difficulty in managing schedules being one of the main barriers to non‐compliance with physical activity. This reflects the importance of health professionals being more aware of the need to adjust therapies to families' routines [31]. Physical activity must be properly incorporated into daily routines so that people with CF, and their parents, feel satisfied with their lives [15]. Patients who incorporate exercise into their routine appear to experience improvements in their physical and mental well‐being, feelings of control, and sense of normality [13].

### 4.1 Limitations

This study acknowledges several limitations due to its exploratory nature. Firstly, variability in interview durations was observed, influenced by participant availability, which may have affected the depth and richness of the data collected. Secondly, the research was conducted within a single hospital context, limiting the generalisability of the findings to other settings and populations. Additionally, the absence of data triangulation through interviews with both children and health professionals presents a limitation, as it restricts a comprehensive understanding of the complexities involved in managing CF from multiple perspectives. Furthermore, there is the possibility that social desirability bias could affect the authenticity of the data collected. To strengthen the validity of our findings, future research should consider the role of developmental transition phases in treatment adaptation and adherence in the context of CF.

Despite these limitations, our study underscores the multifaceted impact of CF on children and parents from parents' perspective, advocating for flexible and personalised care models tailored to families' realities. Collaboration between specialised CF services and public health programmes, alongside technological innovations, can enhance personalised management and adherence. Normalising routines, as per our findings, is crucial for effective family disease management.

## 5 Conclusions

This study underscores the significant emotional and logistical challenges parents face in managing CF in their children. Key findings highlight the importance of early diagnosis and increased parental knowledge in facilitating effective disease management. To enhance clinical practice, healthcare providers should implement tailored educational programmes for families and establish multidisciplinary support teams to address their diverse needs. Additionally, personalised and flexible treatment plans should be developed to accommodate individual family dynamics. Engaging community resources and schools can further support physical activity and overall well‐being for children with CF. These recommendations aim to improve adherence to treatment and, in the end, improve the lives of impacted families.

## Author Contributions

**Sandra Gagulic:** conceptualisation, methodology, data collection, analysis, writing the original draft. **Ana Bártolo:** analysis, review and editing. **Alda Marques:** conceptualisation, methodology, analysis, review and editing, main supervision. **Teresa Reis Silva** and **Raquel Penteado:** review. All authors approved the final version.

## Conflicts of Interest

The authors declare no conflicts of interest.

## Supporting information


**Data S1:** Supporting Information.

## Data Availability

The data that support the findings of this study are available from the corresponding author upon reasonable request.

## References

[1] M. Shteinberg , I. J. Haq , D. Polineni , and J. C. Davies , “Cystic Fibrosis,” Lancet (London, England) 397, no. 10290 (2021): 2195–2211. 34090606 10.1016/S0140-6736(20)32542-3

[2] A. A. Zolin , A. Orenti , A. Jung , and J. van Rens , “ECFSPR Annual Report ” (2021), https://www.ecfs.eu/sites/default/files/Annual%20Report_2021_09Jun2023.pdf.

[3] M. M. Rey , M. P. Bonk , and D. Hadjiliadis , “Cystic Fibrosis: Emerging Understanding and Therapies,” Annual Review of Medicine 70 (2019): 197–210. 10.1146/annurev-med-112717-094536 30312551

[4] A. L. Quittner , E. Saez‐Flores , and J. D. Barton , “The Psychological Burden of Cystic Fibrosis,” Current Opinion in Pulmonary Medicine 22, no. 2 (2016): 187–191. 26814144 10.1097/MCP.0000000000000244

[5] C. Daly , P. Ruane , K. O'Reilly , L. Longworth , and G. Vega‐Hernandez , “Caregiver Burden in Cystic Fibrosis: A Systematic Literature Review,” Therapeutic Advances in Respiratory Disease 16 (2022): 17534666221086416. 35323061 10.1177/17534666221086416 PMC8958690

[6] C. Fitzgerald , S. George , R. Somerville , B. Linnane , and P. Fitzpatrick , “Caregiver Burden of Parents of Young Children With Cystic Fibrosis,” Journal of Cystic Fibrosis 17, no. 1 (2018): 125–131. 29150357 10.1016/j.jcf.2017.08.016

[7] M. B. Happ , L. A. Hoffman , L. W. Higgins , D. Divirgilio , and D. M. Orenstein , “Parent and Child Perceptions of a Self‐Regulated, Home‐Based Exercise Program for Children With Cystic Fibrosis,” Nursing Research 62, no. 5 (2013): 305–314. 23995464 10.1097/NNR.0b013e3182a03503 PMC4053557

[8] N. A. Goodfellow , A. F. Hawwa , A. J. Reid , R. Horne , M. D. Shields , and J. C. McElnay , “Adherence to Treatment in Children and Adolescents With Cystic Fibrosis: A Cross‐Sectional, Multi‐Method Study Investigating the Influence of Beliefs About Treatment and Parental Depressive Symptoms,” BMC Pulmonary Medicine 15 (2015): 43. 25927329 10.1186/s12890-015-0038-7 PMC4417214

[9] A. L. Quittner , J. Abbott , A. M. Georgiopoulos , et al., “International Committee on Mental Health in Cystic Fibrosis: Cystic Fibrosis Foundation and European Cystic Fibrosis Society Consensus Statements for Screening and Treating Depression and Anxiety,” Thorax 71, no. 1 (2016): 26–34. 26452630 10.1136/thoraxjnl-2015-207488 PMC4717439

[10] M. M. Ernst , M. C. Johnson , and L. J. Stark , “Developmental and Psychosocial Issues in CF,” Child and Adolescent Psychiatric Clinics of North America 19, no. 2 (2010): 263–viii. 20478499 10.1016/j.chc.2010.01.004 PMC2874200

[11] C. Snell , S. Fernandes , I. S. Bujoreanu , and G. Garcia , “Depression, Illness Severity, and Healthcare Utilization in Cystic Fibrosis,” Pediatric Pulmonology 49, no. 12 (2014): 1177–1181. 24619910 10.1002/ppul.22990

[12] C. Burtin and H. Hebestreit , “Rehabilitation in Patients With Chronic Respiratory Disease Other Than Chronic Obstructive Pulmonary Disease: Exercise and Physical Activity Interventions in Cystic Fibrosis and Non‐Cystic Fibrosis Bronchiectasis,” Respiration 89, no. 3 (2015): 181–189. 25676797 10.1159/000375170

[13] S. Denford , N. S. Cox , K. A. Mackintosh , et al., “Physical Activity for Cystic Fibrosis: Perceptions of People With Cystic Fibrosis, Parents and Healthcare Professionals,” ERJ Open Research 6, no. 3 (2020): 00294–2019. 32984419 10.1183/23120541.00294-2019 PMC7502697

[14] C. M. Wheatley , B. W. Wilkins , and E. M. Snyder , “Exercise Is Medicine in Cystic Fibrosis,” Exercise and Sport Sciences Reviews 39, no. 3 (2011): 155–160. 21383626 10.1097/JES.0b013e3182172a5a

[15] T. Radtke , S. Smith , S. J. Nevitt , H. Hebestreit , and S. Kriemler , “Physical Activity and Exercise Training in Cystic Fibrosis,” Cochrane Database of Systematic Reviews 8, no. 8 (2022): CD002768. 35943025 10.1002/14651858.CD002768.pub5 PMC9361297

[16] S. Narayanan , J. G. Mainz , S. Gala , H. Tabori , and D. Grossoehme , “Adherence to Therapies in Cystic Fibrosis: A Targeted Literature Review,” Expert Review of Respiratory Medicine 11, no. 2 (2017): 129–145. 28107795 10.1080/17476348.2017.1280399

[17] H. Kimball , T. Douglas , M. Sanders , and V. E. Cobham , “Anxiety in Children With Cystic Fibrosis and Their Parents: A Systematic Review,” Clinical Child and Family Psychology Review 24, no. 2 (2021): 370–390. 33660071 10.1007/s10567-021-00345-5

[18] K. A. Rendle , C. M. Abramson , S. B. Garrett , M. C. Halley , and D. Dohan , “Beyond Exploratory: A Tailored Framework for Designing and Assessing Qualitative Health Research,” BMJ Open 9, no. 8 (2019): e030123. 10.1136/bmjopen-2019-030123 PMC6720470 31462482

[19] World Medical Association , “World Medical Association Declaration of Helsinki: Ethical Principles for Medical Research Involving Human Subjects,” JAMA 310, no. 20 (2013): 2191–2194, 10.1001/jama.2013.281053. 24141714

[20] A. Tong , P. Sainsbury , and J. Craig , “Consolidated Criteria for Reporting Qualitative Research (COREQ): A 32‐Item Checklist for Interviews and Focus Groups,” International Journal for Quality in Health Care 19, no. 6 (2007): 349–357. 17872937 10.1093/intqhc/mzm042

[21] V. Braun and V. Clarke , “Thematic Analysis,” in Encyclopedia of Quality of Life and Well‐Being Research [Internet], ed. F. Maggino (Springer International Publishing, 2020), 1–7, 10.1007/978-3-319-69909-7_3470-2.

[22] H. J. Fuchs , D. S. Borowitz , D. H. Christiansen , et al., “Effect of Aerosolized Recombinant Human DNase on Exacerbations of Respiratory Symptoms and on Pulmonary Function in Patients With Cystic Fibrosis. The Pulmozyme Study Group,” New England Journal of Medicine 331, no. 10 (1994): 637–642. 7503821 10.1056/NEJM199409083311003

[23] Y. S. Lincoln and E. G. Guba , Naturalistic Inquiry (SAGE, 1985), 289–331, 10.1016/0147-1767(85)90062-8.

[24] B. Saunders , J. Sim , T. Kingstone , et al., “Saturation in Qualitative Research: Exploring Its Conceptualization and Operationalization,” Quality & Quantity 52, no. 4 (2018): 1893–1907, 10.1007/s11135-017-0574-8. 29937585 PMC5993836

[25] B. F. Page , L. Hinton , E. Harrop , and C. Vincent , “The Challenges of Caring for Children Who Require Complex Medical Care at Home: ‘The Go Between for Everyone Is the Parent and as the Parent That's an Awful Lot of Responsibility’,” Health Expectations 23, no. 5 (2020): 1144–1154. 32542954 10.1111/hex.13092 PMC7696130

[26] A. Lum , C. E. Wakefield , B. Donnan , M. A. Burns , J. E. Fardell , and G. M. Marshall , “Understanding the School Experiences of Children and Adolescents With Serious Chronic Illness: A Systematic Meta‐Review,” Child: Care, Health and Development 43, no. 5 (2017): 645–662. 28543609 10.1111/cch.12475

[27] T. Thongseiratch and N. Chandeying , “Chronic Illnesses and Student Academic Performance,” Journal of Health Science and Medical Research 38, no. 3 (2020), https://www.jhsmr.org/index.php/jhsmr/article/view/738.

[28] S. C. Bell , M. A. Mall , H. Gutierrez , et al., “The Lancet Respiratory Medicine Commission on the Future of Care of Cystic Fibrosis,” Lancet Respiratory Medicine 8, no. 1 (2020): 65–124. 31570318 10.1016/S2213-2600(19)30337-6 PMC8862661

[29] R. Z. Hayeems , F. A. Miller , C. J. Barg , et al., “Psychosocial Response to Uncertain Newborn Screening Results for Cystic Fibrosis,” Journal of Pediatrics 184 (2017): 165–171.e1. 28279431 10.1016/j.jpeds.2017.01.049

[30] J. E. Dankert‐Roelse and A. V. van Langen , “Newborn Screening for Cystic Fibrosis: Pros and Cons,” Breathe 8, no. 1 (2011): 24–30.

[31] M. D. Barnes , C. L. Hanson , L. B. Novilla , B. M. Magnusson , A. C. Crandall , and G. Bradford , “Family‐Centered Health Promotion: Perspectives for Engaging Families and Achieving Better Health Outcomes,” Inquiry: A Journal of Medical Care Organization, Provision and Financing 57 (2020): 0046958020923537. 32500768 10.1177/0046958020923537 PMC7278332

