# Supplementary material for: Mapping Perceived Impact, Facilitators and Barriers of Cystic Fibrosis Management in Children and Adolescents: A Qualitative Study From the Parents' Perspective
Source: Scand J Caring Sci. 2025 Sep 10;39(3):e70106. doi: 10.1111/scs.70106 (PMC12421296; doi:10.1111/scs.70106)
Supplement: Supplementary file 1 — Data S1: Supporting Information. [file SCS-39-0-s001.docx]

**Report**

**Coding Process [Example]**

| **ID** | **Document** | **Citation Content** | **Codes** | **Reference** |
| --- | --- | --- | --- | --- |
| 1:26 | 711_0016_parents - 6 years old | Yes, yes, they know because they have to give him his medicine every day at noon. Before eating, he has to take the "rionge" so it stays there, for it to take effect. So they also somehow help Diana's situation. With medication. | Adaptation to Disease | 82 - 83 |
| 2:7 | 711_0017_parents_6 years | You treat, you doctors deal with the medication and scientific aspects and the only thing we can do is promote activities that help you physically. | Adaptation to Disease | 86 - 86 |
| 3:15 | 711_0018 parents- 16 years old | We should treat him like a normal child. Ensure he receives the proper care and the extra things he needs to do. We tried to implement that first, and now, as he grows up, we try to ensure he lives a perfectly normal life. | Adaptation to Disease | 5 - 5 |
| 3:17 | 711_0018 parents- 16 years old | No, I think he's even ready. It's the fact that it's something that's always been a part of his life, always; he's never known himself any other way. He can obviously see the differences compared to his sister. She doesn't have any health problems and doesn't have to do all the routines he does. | Adaptation to Disease | 9 - 9 |
| 3:19 | 711_0018 parents- 16 years old | But generally speaking, he complies and goes stipulatedly and goes to the gym twice a week. | Adaptation to Disease | 15 - 15 |
| 3:20 | 711_0018 parents- 16 years old | Yes, yes. He was the one who wanted to go, I wasn't the one who imposed it. Swimming was an imposition. He had to do it somehow. Since he negotiated another solution, we're not going to be fundamentalists here either. | Adaptation to Disease | 19 - 19 |
| 4:13 | 711_0019_parents-11 years old | Dalila takes on everything, does everything and more and is brave. | Adaptation to Disease | 16 - 16 |
| 4:16 | 711_0019_parents-11 years old | It's the mother, yes, it's me. It's like this, she's very responsible. She does everything she's told, sometimes wanting to do more than she can. Because she thinks she can, and we don't tell her no. She knows if she thinks she can, she has to do it, period. Just to make it clear that she has to stop. The rule at home is like this: Dalila, you know that if you're tired, you can't do it. No one forces you to do it. You have to feel if it's enough. So that's how we know we're teaching. | Adaptation to Disease | 24 - 24 |
| 4:17 | 711_0019_parents-11 years old | I trust her with the medication. For example, when she eats at school, she takes it; I know she doesn't hide it. We point out that there are kids who don't take it because they're embarrassed, and because they have their own personality. And she's already been taught that she doesn't need to be embarrassed, that if she's like this, it's because someone wanted her that way. | Adaptation to Disease | 28 - 28 |
| 5:9 | 711_0020_parents-8 years old | She's aware, she is. She's been managing well. She knows she has to take her medication, she knows the precautions she needs to take. | Adaptation to Disease | 39 - 39 |
| 6:7 | 711_0021-parents _10 years | Very well, take him alone. He knows everything. | Adaptation to Disease | 35 - 35 |
| 9:18 | 711_0024- -parents 1-6 years old | It might be better for them, I think they're ready to accept it better (diagnosis in baby) | Adaptation to Disease | 29 - 29 |
| 13:3 | 711_0028_parents_16 years old | She was never a girl who rebelled against the disease, she takes everything very calmly and always with a smile. | Adaptation to Disease | 10 - 10 |
| 13:4 | 711_0028_parents_16 years old | she can already do this self-management | Adaptation to Disease | 23 - 23 |
|  | | | | |
| **ID** | **Document** | **Citation Content** | **Codes** | **Reference** |
| 1:28 | 711_0016_parents - 6 years old | She rides her tricycle inside the house, and her bike only when the weather is nice outside. | Physical Activity | 92 - 92 |
| 1:32 | 711_0016_parents - 6 years old | Yes, yes, I ride a bike, it has space. The house has space, the land has a yard and has space. | Physical Activity | 107 - 107 |
| 2:3 | 711_0017_parents_6 years | What Inês does, she goes to the pool, in the Ílhavo pool with Nuno, I don't know if you know him, but he usually does it. | Physical Activity | 44 - 44 |
| 2:6 | 711_0017_parents_6 years | but she also likes to make rugs | Physical Activity | 85 - 85 |
| 2:8 | 711_0017_parents_6 years | we have the ball that is also used in other sports to work the core, which we still don't have a properly defined exercise for | Physical Activity | 92 - 92 |
| 2:9 | 711_0017_parents_6 years | we let her jump as much as she wants, because when she coughs more it cleans completely, laughs mom | Physical Activity | 97 - 97 |
| 3:18 | 711_0018 parents- 16 years old | Physical exercise, he does physical exercise at school, and he swam for many years, at my unquestionable request. This year, at the beginning of this school year, he asked me to swap swimming for gym. | Physical Activity | 13 - 13 |
| 4:10 | 711_0019_parents-11 years old | She really likes swimming. When she's really sick, I don't let her go; she gets sad. So she really likes it. Physical Education is a five-year-old. So she does it. I was talking to her teacher just a week ago, and she told me she's very pleased with her because she does it because she wants to. Right from the start, I told the teacher she had fibrosis, that she's a child who gets very tired, and I asked her to see if she's a little more tired or a little more infected than not to do it. But she says she doesn't notice any difference between her and the others. And she does exactly the same things. | Physical Activity | 32 - 32 |
| 4:11 | 711_0019_parents-11 years old | It was a choice, it was. There was swimming at school, and she wanted to go because her friends were swimming, and she fell in love with it and really likes it. And then the doctor here also said it was good. Either swimming or ballet or something because they were ready for her to exercise for her lungs. And we thought swimming would be much better, so we put her in swimming, and she likes it. | Physical Activity | 34 - 34 |
| 4:18 | 711_0019_parents-11 years old | She goes to school, she does physical education, she does gymnastics, she does everything the other kids do. She swims, she goes swimming. So she walks, she plays, she runs, she goes to the beach. For now, she's not attached to any machine that makes her different, that makes her feel different, right? | Physical Activity | 28 - 28 |
| 5:12 | 711_0020_parents-8 years old | And when he can, he goes swimming, whenever he can. He goes to the municipal pools for self-recreation, he has a pool card, and whenever he has some free time, he goes swimming. | Physical Activity | 57 - 57 |
| 5:17 | 711_0020_parents-8 years old | She's always loved it. Ever since she was little, she's always been crazy about sports. So kids go through those phases where they say, "I want to be this, I want to be that," and when she was younger, she said she wanted to be a physical education teacher, but then she changed her mind and wanted to be a veterinarian. | Physical Activity | 35 - 35 |
| 5:19 | 711_0020_parents-8 years old | She always wanted to go. So when she was little, she was a soccer fanatic. At school, they called her "Tomboy." Because she was so obsessed with soccer, it was crazy. Then she played federated basketball because she was in a daycare center and they had sports, and she chose basketball. And they played, and she would go play outside, like, at the district level and so on, that was it. But then it ended because the federated teams know they have to have a..., obey certain rules, that there can't be mixed teams, they have age groups, right? | Physical Activity | 51 - 51 |
| 6:8 | 711_0021-parents _10 years | He doesn't like physical activity. It's not that he likes physical activity very much. Of course, I got him into swimming; he already swims at school, and now I've made it a point to have him swim once a week. | Physical Activity | 43 - 43 |
| 6:10 | 711_0021-parents _10 years | And so that's what he's doing. In the summer he gets a lot of exercise because I take him to the fields to farm. | Physical Activity | 47 - 47 |
| 6:21 | 711_0021-parents _10 years | Very good. He even has a treadmill at home. He even likes to run on it. | Physical Activity | 68 - 68 |
| 7:6 | 711_0022_parents -16 years old | do you like running? He loves it. At first it was a problem because we wanted him to swim. | Physical Activity | 11 - 12 |
| 9:12 | 711_0024- -parents 1-6 years old | By bike, he really likes everything | Physical Activity | 48 - 48 |
| 9:14 | 711_0024- -parents 1-6 years old | She likes cycling, because she also does Physical Education at school. | Physical Activity | 56 - 56 |
| 10:6 | 711_0025 parents-16 years old | He likes to play ball. | Physical Activity | 39 - 39 |
| 10:12 | 711_0025 parents-16 years old | Only at school | Physical Activity | 47 - 47 |
| 10:13 | 711_0025 parents-16 years old | OK, and it's because he likes to play football, ride a bike | Physical Activity | 48 - 48 |
| 10:14 | 711_0025 parents-16 years old | In terms of swimming, does it do anything? Also does | Physical Activity | 50 - 51 |
| 11:3 | 711_0026_parents-6 years | He plays ball, jumps, and runs, because he has a backyard, the living room is too big for him to jump, and he rides his bike and runs in the hallway. | Physical Activity | 65 - 65 |
| 13:8 | 711_0028_parents_16 years old | she did ballet for 12 years | Physical Activity | 34 - 34 |
| 14:1 | 711_0029 mother_child- 6 years old | Jump rope | Physical Activity | 3 - 3 |
| 14:2 | 711_0029 mother_child- 6 years old | At home she doesn't stop from jumping, she doesn't stop jumping, she ends up doing exercises in another way but not out of obligation. | Physical Activity | 3 - 3 |
| 14:5 | 711_0029 mother_child- 6 years old | And do you like cycling? Yes. | Physical Activity | 3 - 3 |
| 14:6 | 711_0029 mother_child- 6 years old | She'll play with anything that involves playing and she won't play with anything that's an obligation. | Physical Activity | 3 - 3 |
| 14:8 | 711_0029 mother_child- 6 years old | She goes to ballet because she was advised here by the hospital to do some exercise. | Physical Activity | 3 - 3 |
| 14:10 | 711_0029 mother_child- 6 years old | We're trying to make exercise a little more regular and start to become part of their routine, part of their play. | Physical Activity | 3 - 3 |
| 16:9 | 711_0031 - parents_8 years | Ready to do gymnastics. School gymnastics. | Physical Activity | 32 - 32 |
| 16:10 | 711_0031 - parents_8 years | It's like this, running, jumping rope, cycling, she's been to the pool but was always complaining that the water was cold and then she stopped going there, it was something I wanted her to do because it's good for her. | Physical Activity | 34 - 34 |

| **ID** | **Document** | **Citation Content** | **Codes** | **Reference** |
| --- | --- | --- | --- | --- |
| 2:4 | 711_0017_parents_6 years | when it is really very rough, that the exit from the pool will be a tremendous thermal amplitude, we will not | Barriers to Physical Activity | 47 - 47 |
| 5:20 | 711_0020_parents-8 years old | then there is also the issue of studying, which becomes less available as the course progresses. | Barriers to Physical Activity | 51 - 51 |
| 6:19 | 711_0021-parents _10 years | He likes football, but the problem is that he has to train a lot afterwards. There's a lot of wasted time. | Barriers to Physical Activity | 53 - 53 |
| 6:20 | 711_0021-parents _10 years | And Pedro says he loses a lot in training and then he has to do school work and then it will harm the school. | Barriers to Physical Activity | 53 - 53 |
| 8:8 | 711_0023_parents-17 years old | He was never a child who exercised a lot. | Barriers to Physical Activity | 42 - 42 |
| 8:9 | 711_0023_parents-17 years old | By bike. Now he rides a bike, and these days on vacation he's been riding a bike a lot. But other than that, he could play football, but he doesn't. Walking, I say, let's go for a walk, come with Mom, no. | Barriers to Physical Activity | 45 - 45 |
| 8:11 | 711_0023_parents-17 years old | Activities do not do. | Barriers to Physical Activity | 55 - 55 |
| 8:21 | 711_0023_parents-17 years old | No, I don't think so, I don't think it's because you feel more tired. It is because he himself exercises Lack of interest | Barriers to Physical Activity | 50 - 52 |
| 8:23 | 711_0023_parents-17 years old | This one really likes football, he loves it. Just yesterday he spent the whole afternoon playing football, but Samuel doesn't like to move; he only likes computers. | Barriers to Physical Activity | 73 - 73 |
| 10:15 | 711_0025 parents-16 years old | It's like this, when I also think that he does excessive physical exercise, sometimes blood appears in his secretions and that's why I don't insist too much and I've been taking him to not play ball so much and run so much because I find that when he does excessive physical exercise, his secretions appear with streaks of blood. | Barriers to Physical Activity | 57 - 57 |
| 13:2 | 711_0028_parents_16 years old | It's not easy, she's in her teens now, when she was younger we said "M" let's do the exercises, she did everything right, right, as she grows it's more complicated for her, she does it well when she wants to, she does it badly when she doesn't want to, if she can get by without doing it, that's it. | Barriers to Physical Activity | 10 - 10 |
| 13:9 | 711_0028_parents_16 years old | you don't feel like it and laziness itself | Barriers to Physical Activity | 46 - 46 |
| 13:10 | 711_0028_parents_16 years old | tired more than the others, the little bit that she has wants to be there in her peace | Barriers to Physical Activity | 49 - 49 |
| 14:9 | 711_0029 mother_child- 6 years old | The mother says that she asks to go for a walk at night, but the person wants to get home and rest a little and doesn't feel like doing anything. | Barriers to Physical Activity | 3 - 3 |
| 15:4 | 711_0030 - child_6 years old | No, it's because he has those activities at daycare, but they are paid and I don't have those opportunities to pay. | Barriers to Physical Activity | 2 - 2 |

| **ID** | **Document** | **Citation Content** | **Codes** | **Reference** |
| --- | --- | --- | --- | --- |
| 1:23 | 711_0016_parents - 6 years old | No, no, they're from here. No, that's a lot of money. | Barriers to medication adherence | 35 - 35 |
| 8:7 | 711_0023_parents-17 years old | Badly, badly and he continues to deal badly, badly and he doesn't want to do the flutter, every day a massacre, every day, every day, every day, otherwise when I'm at home in the morning or at night ready because I don't work, Samuel let's do the thing, not now, not now, in a little while Samuel will do this, he'll go, he'll go. He doesn't do anything, I have to force him every day, every day, but it's like that I have to get angry and yell at him to do something and he doesn't do anything at all. | Barriers to Medication Adherence | 39 - 39 |
| 8:12 | 711_0023_parents-17 years old | Speaking of struggles, just doing the flutter every day is a struggle for me. It's not worth doing, oh mom, I'm going to die, why would I do this? | Barriers to Medication Adherence | 82 - 82 |
| 8:13 | 711_0023_parents-17 years old | Even pills, medication, everything when at least he returns to a family member's house or something, he never, always secretly takes the medication, always. | Barriers to Medication Adherence | 100 - 100 |
| 10:4 | 711_0025 parents-16 years old | If you can avoid what you have to do, avoid it, or for example, if you can reject medications, avoid them. | Barriers to Medication Adherence | 33 - 33 |
| 10:10 | 711_0025 parents-16 years old | Because it's boring. It's always the same thing. | Barriers to medication adherence | 35 - 35 |
| 16:16 | 711_0031 - parents_8 years | More or less, then there was also an aspect that I think sometimes I have doubts about, it was also the aspect of her having to take medication at school | Barriers to medication adherence | 18 - 18 |
| 16:17 | 711_0031 - parents_8 years | She talks more about the kids, they make fun of them, even when she wants to cough up the phlegm. Sometimes she swallows it if possible, she's more shy because of these things. | Barriers to medication adherence | 22 - 22 |

| **Document** | **Citation Content** | **Codes** | **Reference** |
| --- | --- | --- | --- |
| 711_0016_parents - 6 years old | She feels a little tired. And if she gets really tired, if she eats, she might throw it all up. And then it doesn't work. She vomits. | Negative Impacts on Children | 98 - 98 |
| 711_0016_parents - 6 years old | She already eats little and with her illness she is not feeling well, even worse. | Negative Impacts on Children | 104 - 104 |
| 711_0017_parents_6 years | Every day she has to do a nebulization with saline solution and then they give us the liquid or serum or sea water to unclog the cavities. | Negative Impacts on Children | 121 - 121 |
| 711_0023_parents-17 years old | It's gotten worse because he thinks he could die any day now. That's what he has in his head. That he won't live for many years. He's been a very rebellious child since elementary school, and now he's at risk of failing his studies. | Negative Impacts on Children | 153 - 153 |
| 711_0028_parents_16 years old | And then we don't have any support at school for her, because it's like this, she misses a week but no one tells her, "M, this is here to make up for you," nothing. The only way we found was tutoring outside. There are subjects she has and can't miss, for example, math. If she misses this or that, she'll never catch the train again. It wasn't the way we found that ended up with her, she also ends up having a more overloaded schedule. | Negative Impacts on Children | 65 - 65 |

| **ID** | **Document** | **Citation Content** | **Codes** | **Reference** |
| --- | --- | --- | --- | --- |
| 1:24 | 711_0016_parents - 6 years old | We lose, at night too. Often at night she's sleeping and suddenly there we go to see something. | Negative Impact on Parents | 41 - 41 |
| 1:25 | 711_0016_parents - 6 years old | Okay, I have to be everywhere. For example, I have a driver's license, so I have to be everywhere. If there's a problem at school, the teacher tells me, and I have to go there. | Negative Impact on Parents | 66 - 66 |
| 1:30 | 711_0016_parents - 6 years old | Yes, yes. The only problem is that she doesn't eat because she has little appetite. People see that she's not eating and get discouraged. | Negative Impact on Parents | 100 - 100 |
| 1:33 | 711_0016_parents - 6 years old | It always affects you; you have to go everywhere and are afraid it will get worse. Not all employers want someone who comes and goes; they have to be there, and it's complicated. | Negative Impact on Parents | 114 - 114 |
| 3:14 | 711_0018 parents- 16 years old | The impact, obviously, as you can imagine, was tremendous, right, as it was with my first child. And it's a serious illness that meant life-changing changes. But like everything in life, you learn to live with it, and I think his doctor at the time also played a very important role in that. | Negative Impact on Parents | 5 - 5 |
| 4:2 | 711_0019_parents-11 years old | So it was a shock for us. It wasn't, and isn't, very well managed, but we have to live with it. There's no one else who, how can I say it, is like this; today we accept it and live with it because she's fine. If we see her even a little worse, we'd be distressed, it's normal. | Negative Impact on Parents | 6 - 6 |
| 4:12 | 711_0019_parents-11 years old | And I did a lot. I told the doctor, "But it can't be done." The girl falls to the side, starts crying, coughing, and falls. "Oh, that's a treat, shake her, call her, and... we went through a traumatic phase until the illness arrived." | Negative Impact on Parents | 10 - 10 |
| 5:16 | 711_0020_parents-8 years old | Sometimes it's complicated. It's not because, I mean, I don't have a job. My husband works, he's a high school teacher. But ever since I was little, when she was born and I was working, he pushed it a little bit on me, like, man, I'm working and you're not, it doesn't make sense, does it? In the meantime, I've worked, and then I stopped working. There are those phases like that. | Negative Impact on Parents | 21 - 21 |
| 7:1 | 711_0022_parents -16 years old | The impact, ready, was diagnosed at 4 months, he was not gaining weight and fundamentally was not growing, the impact was bad. | Negative Impact on Parents | 5 - 5 |
| 8:4 | 711_0023_parents-17 years old | It was horrible, that's it. | Negative Impact on Parents | 17 - 17 |
| 8:14 | 711_0023_parents-17 years old | Since the first day, I almost live only for him, I live for him, it's the morning things, it's the things, uh uh, I'm safe when I'm by his side, I know he takes care of himself, he does everything right, now when I'm not there, that's why I let him go to school and you come home to eat. | Negative Impact on Parents | 132 - 132 |
| 8:27 | 711_0023_parents-17 years old | In tatters. A person wanting, knowing that one day he will have a future, I don't know how he will have it, I don't know, the worst always comes together, isn't it? And seeing this denial of his, not wanting to do things always, everything for his own good, it's only for his own good, I don't do it, I don't do it, he has all day, the bad is only for you, but no, I don't know, look, I don't know. | Negative Impact on Parents | 141 - 141 |
| 11:1 | 711_0026_parents-6 years | I think I've gotten used to it, I don't know. It's only when he has those crises and is hospitalized that I get devastated. | Negative Impact on Parents | 7 - 7 |
| 11:7 | 711_0026_parents-6 years | No. But I'm also taking metalbonchitaopera and an anxiolorzit to sleep and rest better. | Negative Impact on Parents | 9 - 9 |

| **ID** | **Document** | **Citation Content** | **Codes** | **Reference** |
| --- | --- | --- | --- | --- |
| 1:34 | 711_0016_parents - 6 years old | My mother has already passed away, my father is in a nursing home. His parents have already died. So it's really just the two of you. | Family support | 118 - 119 |
| 1:35 | 711_0016_parents - 6 years old | Okay, we always have some friends, but to give me a bike for my son or something, they give it to me but I have to go to work one day... I have friends but at the same time I have to go and pay for it with my work. | Family support | 122 - 122 |
| 6:6 | 711_0021-parents _10 years | Ahhh, we have help. My dad is with them when we're working, he picks them up from school. Everything is more or less under control. | Family Support | 33 - 33 |

| **ID** | **Document** | **Citation Content** | **Codes** | **Reference** |
| --- | --- | --- | --- | --- |
| 2:1 | 711_0017_parents_6 years | I usually have more rigid work schedules for others, my wife, ahah, we chose to do occasional work so that I can be more available for Inês too. | Changes in Routine | 27 - 27 |
| 2:2 | 711_0017_parents_6 years | It's like this, it's a constant struggle, it's not like we have two other daughters at this point, it's no longer a question of, it's a question of, the logistics are already there, sometimes you need imagination and other times you need one or two more people because of the rides, because of the schedules, but we've already adapted to the illness, not to the illness, but to the situation of her needing to go more often than the others and being more available to her in these types of situations. | Changes in Routine | 38 - 38 |
| 3:16 | 711_0018 parents- 16 years old | Yes, obviously. I have to get up earlier to get him on an inhaler, which he has to do before starting his normal daily routine. You have to be careful with the medication he's still taking despite everything, because kids this age... When he was little, he was little, and now he's sixteen, and a person has a lot to think about. Okay, he's sixteen. But, aside from that, I think it impacts our lives. Obviously, when he has to be hospitalized, it has a big impact. Fortunately, nothing happened most of the time. | Changes in Routine | 7 - 7 |
| 4:14 | 711_0019_parents-11 years old | Well, that's it, I won't deny that my life is completely dedicated to Dalila. | Changes in Routine | 18 - 18 |
| 4:15 | 711_0019_parents-11 years old | I thought it was an option. Because I worked and had my own practice—I'm a beautician, and I had my own practice, but when they told me that if I... from now on, I'd have to spend three months at home, one in the hospital, there's no one who can keep a business open. It's not. As it happened immediately, out of shock, exactly that, I considered stopping. When I stopped, well, since I'm fortunate enough to have a lot of clients, I continued to do home visits. So, I stopped having an open-door business because I thought that to have an open-door business, I couldn't stop being there. | Changes in Routine | 20 - 20 |
| 5:15 | 711_0020_parents-8 years old | But there was a period when the hospital allowed us to bring three months' worth of medication. But then, after a certain point, they banned it. Which means I have to come here during breaks to pick up the medication, so I don't have to ask favors from A, B, and C. | Changes in Routine | 19 - 19 |
| 6:4 | 711_0021-parents _10 years | Well, definitely to come here to the hospital, otherwise I wouldn't come here, right? If I didn't have a problem, I wouldn't come to Coimbra. | Changes in Routine | 25 - 25 |
| 6:5 | 711_0021-parents _10 years | I'm forced to adjust; my job can't. I always have to comply. To come to appointments, I always have to adjust my work. Now it's always more complicated. The situation is, you have to be more close. It's not. You have to be present. A person is more vigilant than not having problems. And those little things always. A more attentive person. | Changes in Routine | 29 - 29 |
| 7:3 | 711_0022_parents -16 years old | Fortunately not because coming here upsets, but it doesn't upset that much. | Changes in Routine | 10 - 10 |
| 8:17 | 711_0023_parents-17 years old | It's like this, there weren't many changes, there wasn't any change, change, everything became more, we continue to do what we did but psychologically. | Changes in Routine | 36 - 36 |
| 9:5 | 711_0024- -parents 1-6 years old | It changed because I was working and I had to leave work because they didn't want to take care of it in the garden next to the thing because of the medication and all those things. Then I had to stop working, talking about it, it got a little complicated, but oh well. | Changes in Routine | 19 - 19 |
| 13:5 | 711_0028_parents_16 years old | Yes, it is very homogeneous in that field and it is not that situation where we always have to play the lottery, no, it is very predictable and it is very easy | Changes in Routine | 27 - 27 |
| 13:7 | 711_0028_parents_16 years old | It always ends up affecting us. It's easy for us, for example, for him to be with her. I try to be absent as little as possible, but... we try to manage it that way, but when necessary, she's also becoming more and more independent, and we're trying to do that too. Because whether we like it or not, we want her to have a perfectly normal life, and next year she'll go to college. We ourselves are trying to cut back a bit. Well, despite her father, he's going to work, and since he works close to home, he goes to see her at lunch when she's sick and checks on her and tries to manage it that way, but without having to stay... Only on occasions when she has a high temperature and shortness of breath, and that's it's convenient for someone to be with her, but if it's just coughing up the cough. For example, she's in a crisis now and she hasn't had a fever because she started intensifying the kinesiotherapy so much that she managed to keep it down. And we're trying to do that. | Changes in Routine | 32 - 32 |
| 16:15 | 711_0031 - parents_8 years | I came at night, I always spent the weekends here, all nights and that all changed a bit, it's also tiring, we're not very far from here but we're not close either, the trips are not very accessible for the means | Changes in Routine | 16 - 16 |
